# Comparative efficacy and safety of intravenous ferric carboxymaltose and iron sucrose for iron deficiency anemia in obstetric and gynecologic patients: A systematic review and meta-analysis

**DOI:** 10.1097/MD.0000000000024571

**Published:** 2021-05-21

**Authors:** Hye Won Shin, Doo Yeon Go, Suk Woo Lee, Yoon Ji Choi, Eun Ji Ko, Hae Sun You, Yoo Kyung Jang

**Affiliations:** Department of Anesthesiology and Pain Medicine, College of Medicine, Korea University Anam Hospital, Seoul, Republic of Korea.

**Keywords:** ferric carboxymaltose, gynecologic, iron deficiency anemia, iron sucrose, obstetric

## Abstract

**Introduction:**

Iron deficiency anemia (IDA) is common among obstetric and gynecologic patients. This systematic review aimed to assess the comparative efficacy and safety of commonly used intravenous (IV) iron formulations, ferric carboxymaltose (FCM), and iron sucrose (IS) in the treatment of IDA in obstetric and gynecologic patients.

**Methods:**

We systematically searched PubMed, EMBASE, Cochrane CENTRAL, and Google Scholar for eligible randomized controlled trials (RCTs) comparing IV iron replacement using FCM and IS up to October 2019. The primary outcome was to compare the efficacy of FCM and IS, assessed by measuring serum hemoglobin (Hb) and ferritin levels before and after iron replacement. The secondary outcome was to compare the safety of FCM and IS, assessed by the incidence of adverse events during iron replacement. The meta-analysis was performed using RevMan 5.3.

**Results:**

We identified 9 RCTs with 910 patients (FCM group, n = 456; IS group, n = 454). Before iron replacement, FCM and IS group patients had similar baseline Hb (mean difference [MD], 0.04 g/dL; 95% confidence interval [CI], −0.07 to 015; *I*^2^ = 0%; *P* = 0.48) and ferritin levels (MD, −0.42 ng/mL; 95% CI, −1.61 to 0.78; *I*^2^ = 45%; *P* = 0.49). Following iron replacement, patients who received FCM had higher Hb (MD, 0.67; 95% CI, 0.25–1.08; *I*^2^ = 92%; *P* = 0.002) and ferritin levels (MD, 24.41; 95% CI, 12.06–36.76; *I*^2^ = 75%; *P* = 0.0001) than patients who received IS. FCM group showed a lower incidence of adverse events following iron replacement than IS group (risk ratio, 0.53; 95% CI, 0.35–0.80; *I*^2^ = 0%; *P* = 0.003). Serious adverse events were not reported in any group.

**Conclusion:**

FCM group showed better efficacy in increasing Hb and ferritin levels and a favorable safety profile with fewer adverse events compared with IS group for IDA treatment among obstetric and gynecologic patients. However, this meta-analysis was limited by the small number of RCTs and high heterogeneity.

**Trial registration:**

The review was prospectively registered with the International Prospective Registry of Systematic Reviews (https://www.crd.york.ac.uk/prospero/, registration number CRD42019148905).

## 1 Introduction

Iron deficiency anemia (IDA) is common among women. In women of childbearing age, the most common cause of IDA is loss of iron due to menstrual blood loss or pregnancy. The risk factors related to IDA among women include low socio-economic status, social deprivation, teenage pregnancy, high parity, multiple pregnancies, and short inter-pregnancy intervals.^[1]^

IDA is the final state of iron depletion due to inability to maintain physiologic balance of iron uptake and utilization.^[2]^ Iron deficiency depresses the erythropoietic system resulting in decreased hemoglobin (Hb) levels.^[3]^ Anemia in women is defined by the World Health Organization as an Hb level < 12 g/dL in nonpregnant women and < 11 g/dL in pregnant women.^[4]^ The prevalence of IDA among pregnant women increases from 6.9% in the first trimester to 14.3% and 28.4% in the second and third trimesters, respectively.^[5]^

Traditionally, oral iron replacement is used as the first-line therapy in patients with IDA due to its ease of administration, and early initiation of this treatment can correct anemia. However, oral iron has some disadvantages such as side-effects, poor compliance, and limited gastrointestinal absorption.^[6,7]^ Sometimes, oral iron replacement even after prolonged treatment may fail to adequately correct anemia and iron reserves, before delivery in pregnant women.^[7,8]^ This situation can be easily and efficiently remedied by the use of intravenous (IV) iron replacement, which can deliver the total amount of required iron over a short period. Currently, the most commonly used IV iron formulations are iron sucrose (IS) and ferric carboxymaltose (FCM).^[1,9–13]^ To date, there have been no systematic reviews comparing IV iron formulations, such as FCM and IS, in the treatment of IDA among obstetric and gynecologic patients.

Therefore, this study aimed to perform a systematic review and meta-analysis of randomized controlled trials (RCTs) comparing the efficacy and safety of most commonly used IV iron formulations, FCM and IS, in the treatment of IDA among obstetric and gynecologic patients.

## 2 Materials and methods

In this systematic review and meta-analysis of clinical trials, we evaluated the comparative efficacy and safety of IV FCM and IS in the treatment of IDA among obstetric and gynecologic patients. This analysis was conducted according to the guidelines of the Cochrane Handbook for Systematic Reviews of Interventions,^[14]^ and was reported based on the Preferred Reporting Items for Systematic Reviews and Meta-analyses statement.^[15]^ The review was prospectively registered with the International Prospective Register of Systematic Reviews (https://www.crd.york.ac.uk/prospero/, registration number CRD42019148905). This study did not require ethical approval since all analyses were based on previously published RCTs.

### 2.1 Literature search

We systematically searched the databases, including PubMed, EMBASE, Cochrane CENTRAL, and Google Scholar, to retrieve clinical trials involving adults (older than 18 years), which were published up to October 2019, with no language restrictions. As outlined in the Supplementary Information (S1), the key words used during the search were “intravenous,” “ferric carboxymaltose,” “iron sucrose,” “obstetric,” “gynecologic,” and “randomized.”

### 2.2 Study selection

Peer-reviewed RCTs that evaluated the comparative efficacy and safety of IV FCM and IS in the treatment of IDA among obstetric and gynecologic patients were included in our analysis. Review articles, observational studies, case reports, letters to the editor, commentaries, proceedings, laboratory studies, and other nonrelevant studies were excluded. Two authors (DYG and YJC) independently assessed the articles for compliance with the inclusion/exclusion criteria. Disagreements were resolved by discussion or consultation with a third independent investigator (HWS).

### 2.3 Data extraction and assessment of outcomes

Several studies have assessed the efficacy of iron replacement therapy in patients with IDA by measuring the serum Hb and ferritin levels. Therefore, in our systematic review, the primary outcome was to compare the efficacy of IV FCM and IS by measuring serum Hb and ferritin levels before and after iron replacement. The secondary outcome was to compare the safety of IV FCM and IS based on the incidence of adverse events that occurred during iron replacement. We also compared the quality of life (QoL) and fatigue levels between the FCM and IS groups. Using standardized forms, two authors (HSY and SWL) extracted the following data independently: the name of the first author, year of publication, clinical inclusion criteria, country, number of patients who received IV iron replacement, serum Hb and ferritin levels, adverse events, and data on parameters for QoL and fatigue levels. The control group (IS group) included patients who received IV IS, while the intervention group (FCM group) included patients who received IV FCM as iron replacement therapy.

### 2.4 Assessment of bias risk

Two authors (YKJ and EJK) independently assessed the quality of the clinical trials. We used the Cochrane risk-of-bias tool to assess the quality of RCTs considering the following 7 potential sources of bias: random sequence generation, allocation concealment, blinding of participants, blinding of outcome assessment, incomplete outcome data, selective outcome reporting, and others.

### 2.5 Statistical analysis

Statistical analyses were performed using RevMan 5.3 (Cochrane Collaboration, London, UK). Mean differences (MDs) with their 95% confidence intervals (CIs) were calculated for continuous variables, and risk ratios (RR) with their corresponding 95% CIs were obtained for dichotomous outcome measures. The overall data were compared using a Z-test. All reported *P* values are 2-sided, and *P* values < 0.05 were considered statistically significant. Statistical heterogeneity was estimated using the *I*^2^ statistic, which was considered significant for *I*^2^ values > 50%. The Mantel–Haenszel or inverse variance fixed-effects model was used for the study without significant heterogeneity, while the Mantel–Haenszel or inverse variance random-effects model was used for the study with significant heterogeneity. To assess the heterogeneity of the outcomes, we performed a sensitivity analysis by sequentially excluding one study in each turn to examine the influence of a single study on the overall estimate. We also performed a subgroup analysis of posttreatment Hb and ferritin levels according to patient types (obstetric, gynecologic, and obstetric and gynecologic). Funnel plots were used to assess the publication bias if more than 10 RCTs were included in a comparison.^[14]^

## 3 Results

### 3.1 Literature search

**Figure 1:**
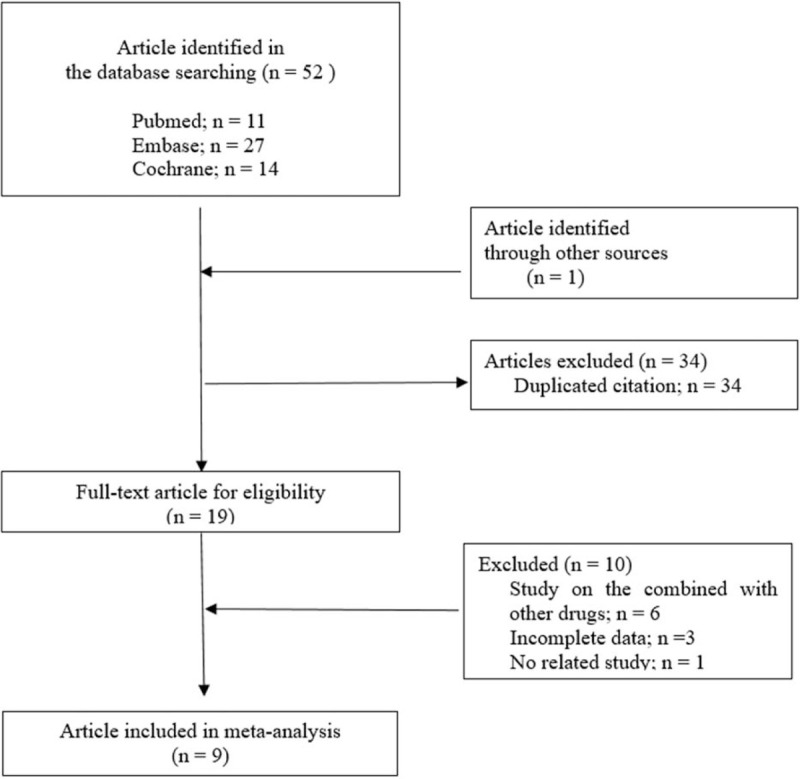
PRISMA flow diagram for inclusion and exclusion of trials.

During the initial electronic search, 53 potential clinical trials were identified (11 from PubMed, 27 from EMBASE, 14 from Cochrane CENTRAL, and 1 from other sources), as described in Figure 1. We identified 9 RCTs^[16–24]^ that compared the efficacy and safety of IV FCM and IS in the treatment of IDA among obstetric and gynecologic patients; these studies were published between 2015 and 2019.

### 3.2 Study characteristics and data

In the 9 RCTs, a total of 910 patients received IV iron replacement for treatment of IDA (IV FCM, n = 456; IV IS, n = 454). The characteristics of the RCTs included in our meta-analysis are shown in Table 1.^[16–26]^ Eight studies^[17–24]^ were conducted in India, and 1 study^[16]^ was conducted in the Republic of Korea (Table 1). The patients with IDA included in this systematic review were obstetric patients including pregnant women^[17]^ and postpartum women,^[19,21–24]^ gynecologic patients including women with menorrhagia,^[16,20]^ and combined obstetrics-gynecologic patients including women with pregnancy, postpartum issues, menorrhagia, uterine bleeding, ovarian cysts, and ligation^[18]^ admitted for treatment of IDA.

**Table 1:** Study characteristics of the RCTs included in the meta-analysis.

Studies (first author, date of publication)	Clinical inclusion criteria	The presence and the degree of bleeding	Sample size	Intravenous iron dosage	Total intravenous dose of iron	Time of Hb, ferritin assessment after treatment initiation	Quality of life
Lee 2019^[16]^	Women with IDA due to menorrhagia (Hb < 10 g/dL, ferritin < 30 ng/mL)	Menorrhagia with heavy cyclical menstrual blood loss (>80 mL per cycle) over 3 cycles	FCM group, n = 52	FCM: body weight < 50 kg (500 mg iron), > 50 kg (1000 mg iron), single dose	FCM: 923.1 ± 207.3 mg (at single visit)	2 wk	Short Form-12 Health Survey (SF-12), no differences in outcomes between groups.
			IS group, n = 49	IS: Ganzoni formula. (200 mg/wk × 3 wk)	IS: 939.1 ± 352.3 mg (over 3–8 visits)		
Jose 2019^[17]^	Pregnant women with IDA (Hb > 6.0 g/dL, < 10 g/dL)	No active bleeding	FCM group, n = 50	FCM: Ganzoni formula. (1000 mg single dose, repeated after days 7 and 14)	FCM: 1739.6 ± 105.5 mg (over 2 wk)	3, 6^∗^, 12 wk	Fatigue (LASA; Linear Analogue Scale Assessment), lower in the FCM group compared with the IS group (*P* = 0.0048).
			IS group, n = 50	IS: Ganzoni formula. (300 mg twice/wk)	IS: 1,730.4 ± 121.9 mg (over 3 wk)		
Naqash 2018^[18]^	Obstetric (pregnancy, postpartum) and gynecologic (menorrhagia, uterus heavy bleeding, ovarian cyst) patients with IDA	Menorrhagia (bleeding for more than 7 d with heavy clots, and doubling of pads or tampons to manage menstrual flow), Uterus heavy bleeding (more than 12 d with doubling of pads or tampons to manage menstrual flow)	FCM group, n = 100	FCM: Ganzoni formula (1000 mg single dose)	FCM: no details provided.	2, 4^∗^ wk	Short Form 36 (SF-36), improved outcomes in the FCM group compared with the IS group (no comments for *P* value).
			IS group, n = 100	IS: Ganzoni formula (200 mg × 2–3 times)	IS: no details provided.		
Sumathy 2017^[19]^	Postpartum women with IDA (Hb 8–10 g/dL)	Normal postpartum women at 24 h after delivery	FCM group, n = 50	FCM: modified Ganzoni formula (single dose)	FCM: 937.94 mg	2, 4^∗^ wk	No details provided
			IS group, n = 50	IS: modified Ganzoni formula (200 mg/d × 5 times)	IS: 935.53 mg		
Mahey 2016^[20]^	Women older than 18 years with heavy menorrhagia and IDA (Hb 6.0–10.9 g/dL)	Menorrhagia with a pictorial bleeding assessment chart score higher than 100	FCM group, n = 30	FCM: modified Ganzoni formula (1000 mg single dose)	FCM: no details provided	1, 6^∗^, 12 wk	Fatigue (LASA; Linear Analogue Scale Assessment), improved outcomes in both groups without any statistical differences
			IS group, n = 30	IS: modified Ganzoni formula (300 mg twice weekly)	IS: no details provided		
Joshi 2016^[21]^	Postpartum women with IDA (Hb ≤ 6.0 g/dL, ≥ 11 g/dL)	Postpartum women with significant vaginal bleeding in 24 h prior was excluded	FCM group, n = 90	FCM: 1000 mg single dose	FCM: 1000 mg	30 d	No details provided
			IS group, n = 83	IS: 200 mg × 5 times	IS: 1000 mg		
Hol 2015^[22]^	Postpartum women with IDA (Hb, 7–11 g/dL)—moderate anemia group	Postpartum women suffering from acute blood loss was excluded	FCM group, n = 20	FCM: 500 mg × 2 times	FCM, 500 mg	1 d, 6^∗^ wk	No details provided
			IS group, n = 20	IS: 200 mg × 4 times	IS, 500 mg		
Garg 2015^[23]^	Postpartum women with IDA (Hb < 7 g/dL)	All normal delivery and cesarean delivery patient	FCM group, n = 50	FCM: body weight (kg) × [target Hb (11 g/dL) – actual Hb (g/dL)] × 0.24 + storage iron (500 mg), single dose	FCM: no details provided	2, 4^∗^ wk	No details provided
			IS group, n = 50	IS: body weight (kg) × [target Hb (11 g/dL) —actual Hb (g/dL)] × 0.24 + storage iron (500 mg), 200 mg × 5 times	IS: no details provided		
Rathod 2015^[24]^	Postpartum women with IDA (Hb < 10 g/dL)	No details about the bleeding	FCM group, n = 14	FCM: body weight (kg) × [target Hb (12 g/dL) —actual Hb (g/dL)] × 0.24 + storage iron (500 mg), single dose	FCM: no details provided	2, 6^∗^ wk	General Well-Being Scale, higher scores in the FCM group compared with the IS group
			IS group, n = 22	IS: body weight (kg) × [target Hb (11 g/dL) —actual Hb (g/dL)] × 0.24 + storage iron (500 mg), 200 mg × 5 times	IS: no details provided		

### 3.3 Assessment for the risk of bias

**Figure 2:**
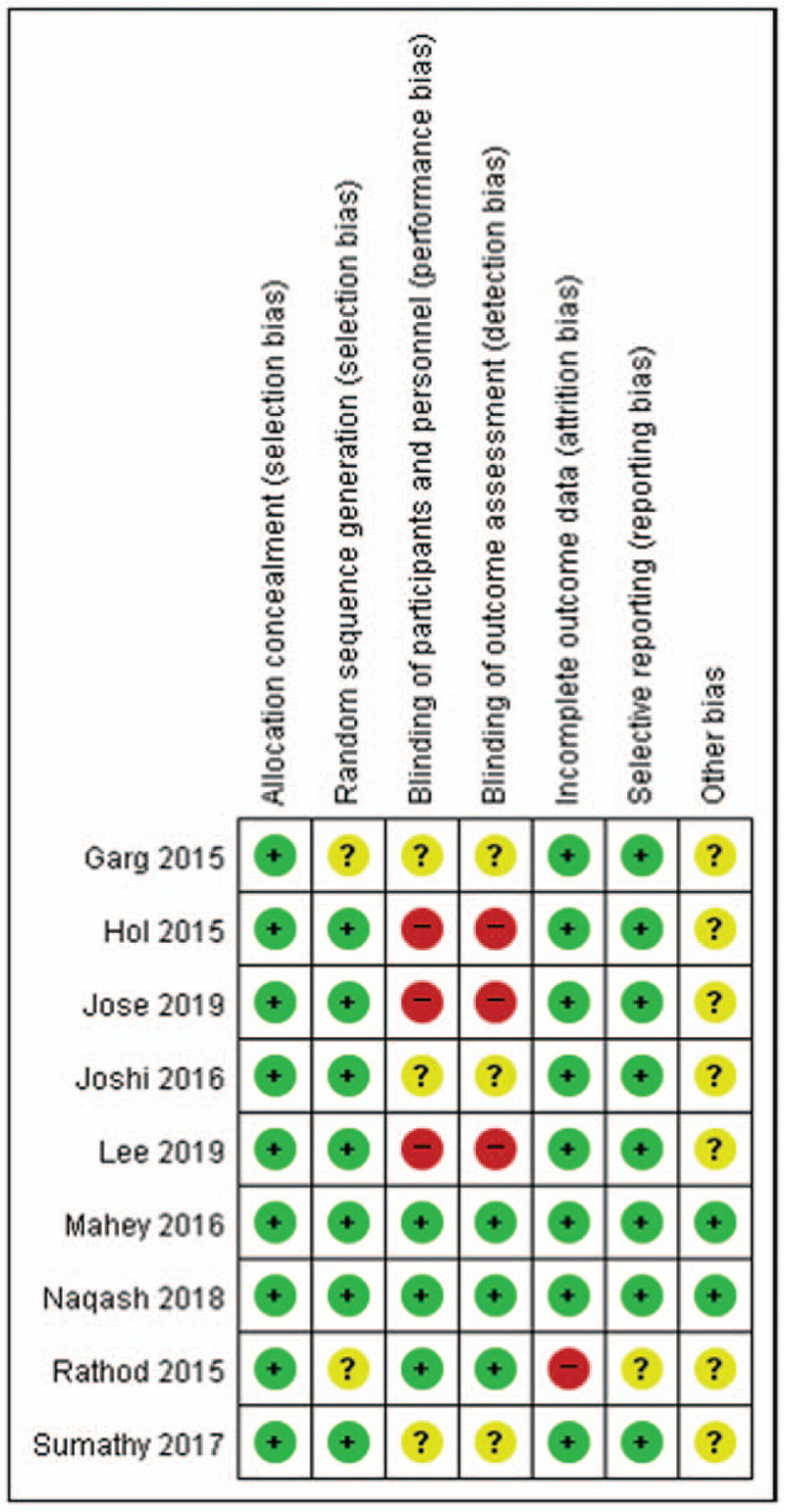
Assessment for the risk of bias reflecting the quality of the included trials. The “plus sign” indicates a low risk of bias; the “minus sign” indicates a high risk of bias; and the “question mark” indicates an unclear risk of bias.

The results of our assessment for the risk of bias regarding the quality of the included studies are summarized in Figure 2. All the studies described allocation concealment. However, 3 RCTs showed a high risk of bias, specifically due to the absence of blinding of participants or outcome assessments.^[16,17,22]^ All other categories of bias were assessed as low risk or “no provided details” for the included studies.

### 3.4 Publication bias

Because the number of included studies was not more than 10 for a certain outcome, we could not evaluate the existence of publication bias by the inspection of funnel plots.^[14]^

### 3.5 Result of meta-analysis

#### 3.5.1 Comparison of serum hemoglobin levels

**Figure 3:**
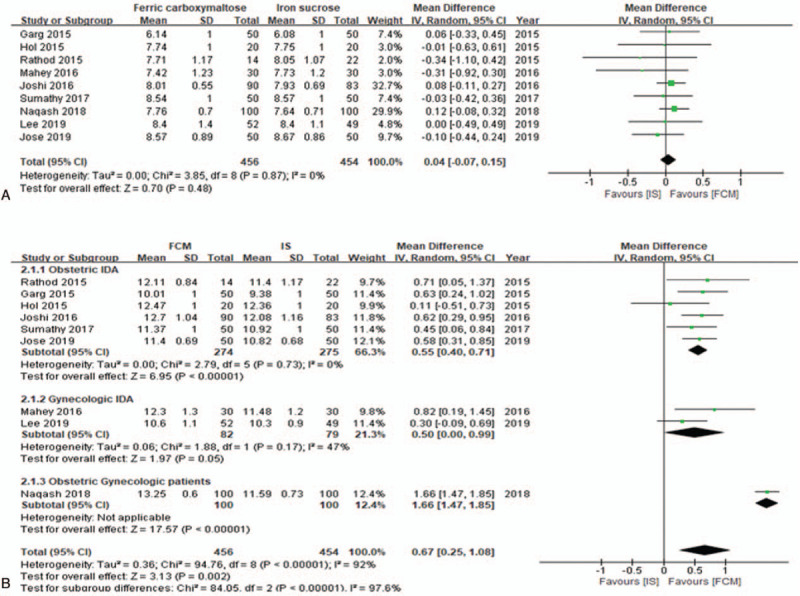
Forest plot demonstrating the comparison of serum hemoglobin levels (g/dL) between groups. (A) Comparison of serum hemoglobin levels before intravenous iron replacement and (B) after intravenous iron replacement. CI = confidence interval, FCM = ferric carboxymaltose, Hb = hemoglobin, *I*^2^ = heterogeneity, IS = iron sucrose.

Nine studies^[16–24]^ with a total of 910 patients provided data on the changes in Hb level (g/dL). Prior to iron replacement therapy, baseline Hb levels were similar in the FCM and IS groups (MD, 0.04 g/dL; 95% CI, −0.07 to 015; *I*^2^ = 0%; *P* = 0.48) (Fig. 3A). Following IV iron replacement, Hb levels were higher among patients who received IV FCM compared with those who received IV IS (MD, 0.67; 95% CI, 0.25–1.08; *I*^2^ = 92%; *P* = 0.002) (Fig. 3B). The measurement of Hb levels during follow-up was at the following time points: after 2 weeks,^[16]^ 4 weeks,^[18,19,21,23]^ and 6 weeks^[17,20,22,24]^ of initiation of IV iron replacement therapy (Table 1). In the sensitivity analysis, when we excluded the study by Naqash et al,^[18]^ the heterogeneity (*I*^2^) of the included studies decreased markedly from 92% to 0%, and the meta-analysis showed higher significance of the posttreatment Hb levels in the FCM group than in the IS group (MD, 0.53; 95% CI, 0.39–0.68; *I*^2^ = 0%; *P* < 0.00001). We performed a subgroup analysis of posttreatment Hb, according to the type of patients (obstetric, gynecologic, and combined obstetric and gynecologic). In all subgroups, posttreatment Hb levels were significantly higher among patients who received IV FCM compared with those who received IV IS (obstetric patients: MD, 0.55 g/dL; 95% CI, 0.40–0.47; *P* < 0.00001; gynecologic patients: MD, 0.50 g/dL; 95% CI, 0.00–0.99; *P* = 0.05; obstetric and gynecologic patients: MD, 1.66 g/dL; 95% CI, 1.47–1.85; *P* < 0.00001).

#### 3.5.2 Comparison of serum ferritin levels

**Figure 4:**
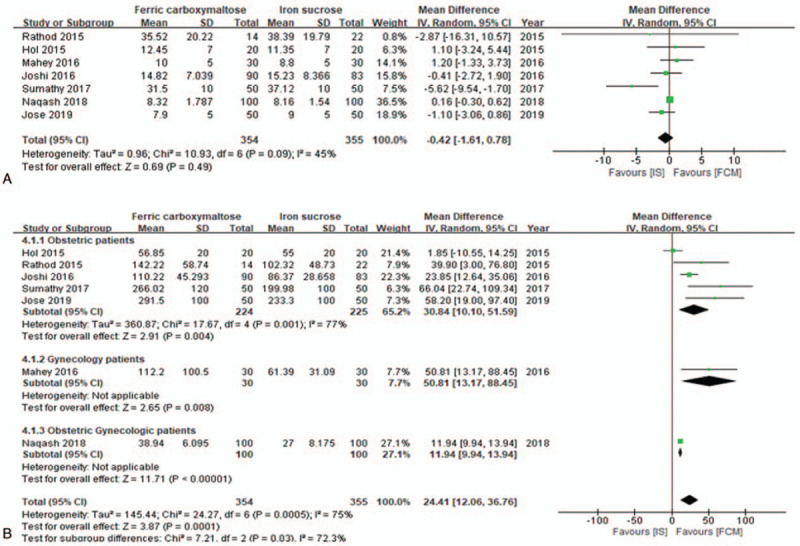
Forest plot demonstrating the comparison of serum ferritin (ng/mL) levels between groups. (A) Comparison of serum ferritin levels before intravenous iron replacement and (B) after intravenous iron replacement. CI = confidence interval, FCM = ferric carboxymaltose, *I*^2^ = heterogeneity, IS = iron sucrose.

Seven studies^[17–22,24]^ with a total of 709 patients provided data on the changes in ferritin level (ng/mL). Prior to IV iron replacement therapy, the FCM and IS groups had similar baseline ferritin levels (MD, −0.42 ng/mL; 95% CI, −1.61 to 0.78; *I*^2^ = 45%; *P* = 0.49) (Fig. 4A). Following IV iron replacement, patients who received IV FCM had higher ferritin levels than the patients who received IV IS (MD, 24.41 ng/mL; 95% CI, 12.06–36.76; *I*^2^ = 75%; *P* = 0.0001) (Fig. 4B). The ferritin levels were measured during follow-up at the following time points: after 4 weeks^[18,19,21,23]^ and 6 weeks^[17,20,22,24]^ of initiation of IV iron replacement therapy. The sensitivity analysis did not yield a change in the overall significance for the comparison of serum levels between the FCM and IS groups. We also performed a subgroup analysis of posttreatment ferritin levels based on the types of patients (obstetric, gynecologic, and combined obstetric and gynecologic). In all subgroups, posttreatment ferritin values were significantly higher among patients who received IV FCM than among those who received IV IS (obstetric patients: MD, 30.84 ng/mL; 95% CI, 10.10–51.59; *I*^2^ = 77%; *P* = 0.004; gynecologic patients: MD, 50.81 ng/mL; 95% CI, 13.17–88.45; *P* = 0.008; obstetric and gynecologic patients: MD, 11.94 ng/mL; 95% CI, 9.94–13.94; *P* < 0.00001).

### 3.6 Adverse events according to the intravenous iron formulation (number of events/person)

**Figure 5:**
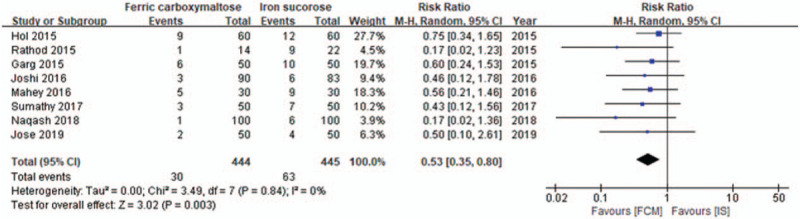
Forest plot showing the incidence of adverse events (number of events/person) between groups. CI = confidence interval, FCM = ferric carboxymaltose, *I*^2^ = heterogeneity, IS = iron sucrose.

Eight studies^[17–24]^ provided data regarding adverse events according to the formulation used in IV iron replacement. The FCM group had a lower incidence of adverse events than the IS group (RR, 0.53; 95% CI, 0.35–0.80; *I*^2^ = 0%; *P* = 0.003) (Fig. 5). The sensitivity analysis for the comparison of adverse events between groups did not yield a change in the overall result. We also recorded the adverse effects reported in the 2 groups in the included studies (Table 2). Hypophosphatemia was reported in 2 studies.^[17,20]^ No serious adverse events were reported in the included studies.

**Table 2:** Adverse effects in the included studies according to the formulation of intravenous iron.

Studies (first author, date of publication)	Iron formulation administered	Adverse events (number of patients)
Lee 2018	FCM and IS	Headache (4, no details for the included group)
Jose 2019	FCM	Injection site reaction (1), elevated serum transaminase level (1), hypophosphatemia (2)
	IS	Injection site reaction (2), epigastric pain (2), hypophosphatemia (3)
Naqash 2018	FCM	Headache (1)
	IS	Nausea and tingling sensation (3), headache (2), arthralgia (1)
Sumathy 2017	FCM	Nausea, giddiness, arthralgia (total 3)
	IS	Nausea, vomiting, urticaria (total 7)
Mahey 2016	FCM	Gastritis (2), itching (2), rash (1), hypophosphatemia (15)
	IS	Fever (2), gastritis (2), vomiting (2), injection site pain (3), itching (1), hypophosphatemia (12)
Joshi 2016	FCM	Chest discomfort (2), nausea (1)
	IS	Injection site reaction, nausea, hypotension (total 6)
Garg 2015	FCM	Injection site reaction (1), rash (1), headache (3), nausea (1)
	IS	Injection site reaction (2), rash (2), headache (4), nausea (2)
Hol 2015	FCM	Rigors (1), headache (2), flushing or skin eruption (1), itching (2), injection site pain or redness (3)
	IS	Rigors (1), headache (1), itching (3), injection site pain or redness (5), nausea and vomiting (2)
Rathod 2015	FCM	Arthralgia or tingling sensation (1)
	IS	Transient hypotension (3), arthralgia or tingling sensation (3)

### 3.7 QoL and fatigue levels according to the formulation of intravenous iron therapy

QoL and fatigue levels were reported in 5 RCTs^[16–18,20,24]^ using diverse measurements methods such as a Short Form-12 Health Survey,^[16]^ Short Form-36 Health Survey,^[18]^ Linear Analogue Scale Assessment,^[17]^ and Scale of General Well-Being.^[24]^ Regarding QoL and fatigue levels, 4 studies^[16–18,24]^ showed favorable outcomes with the FCM group outcomes being more favorable than the IS group.

## 4 Discussion

In the present meta-analysis, we found that IV FCM was superior compared with IV IS as an iron replacement agent for the treatment of IDA among obstetric and gynecologic patients. IV FCM yielded higher Hb and ferritin levels, as well as demonstrated a more favorable safety profile with fewer adverse events among obstetric and gynecologic patients with IDA compared with IV IS.

IDA is widespread worldwide, but mostly overlooked. IDA primarily results in fatigue, poor work capacity, diminished QoL, and even infertility in women.^[27]^ IDA in pregnancy may lead to preterm labor, low birth weight, and perinatal maternal mortality.^[27]^ Therefore, active treatment of IDA among obstetric and gynecologic patients is very important in the prevention of morbidity and mortality of women.^[28]^

Oral iron is a convenient mode of iron replacement. However, oral iron is insufficient for the treatment of IDA, particularly in the third trimester of pregnancy.^[8]^ Oral iron may take 3 to 6 months to replete body iron stores and normalize the ferritin levels.^[29]^ In a study on the dosage of oral iron (1 or 2 capsules/d) in the treatment of IDA during pregnancy, there were no differences in Hb and ferritin levels based on the oral iron dose.^[30]^ This might be due to increased hepcidin level caused by frequent oral iron intake in high doses. Hepcidin regulates iron homeostasis and inhibits iron absorption for up to 24 hours after the last administration of oral iron formulation.^[31]^

The Network for the Advancement of Patient Blood Management, Haemostasis, and Thrombosis recommends consideration of IV iron in pregnant women who fail to respond to oral iron supplementation within 2 to 4 weeks of initiation of therapy, those with severe anemia (Hb < 8.0 g/dL), or newly diagnosed IDA beyond 34 weeks of gestation.^[32]^ In general, IV iron formulations resulted in adverse effects such as hot flushes, chest tightness, headache, nausea, vomiting, mild fever, arthralgia, and anaphylaxis caused by the toxic reaction of free iron. High molecular weight iron dextran formulations also demonstrated concerning adverse effects, specifically, anaphylactic reaction, and circulatory collapse.^[33,34]^

FCM and IS are dextran-free IV iron formulations that are commonly used for IDA. IS has been the standard formulation used in IDA treatment for the past 20 years.^[17]^ It has few adverse events and requires no test dose. The disadvantages of IS are the necessity of multiple infusions and prolonged infusion time (at a dose of up to 300 mg per dose or 600 mg per week). FCM is the newest iron formulation approved by the Food and Drug Administration in 2013. FCM can be administered at large iron doses as a single infusion over 15 minutes (usual doses of 750 mg in the USA and up to 1000 mg in the European Union).^[35]^ The efficacy and tolerability of FCM have been validated in the treatment of IDA in patients with irritable bowel disease,^[36]^ and most recently in chronic heart failure.^[37]^

In our meta-analysis, posttreatment Hb and ferritin levels were higher in FCM group compared with the IS group. In a previous observational study on 863 pregnant women, Froessler et al^[38]^ documented significantly increased Hb and ferritin levels after IV FCM treatment. In the studies conducted by Lee et al and Jose et al, FCM led to a significantly higher and rapid increase in the Hb levels compared with IS, following significantly fewer treatment sessions.^[16,17]^ In the sensitivity analysis of posttreatment Hb levels in our study, heterogeneity (*I*^2^) among the studies was decreased to 0% when we excluded the study by Naqash et al.^[18]^ This may be attributed to differences in the study population; the study by Naqash et al included a mixed group of obstetric and gynecologic patients in variable disease states, whereas the other studies included either obstetric or gynecologic patients alone.

The advantage of using FCM is the prompt correction of IDA.^[16,24,39]^ The treatment time required to administer a 1000 mg dosage of iron was a single day in the FCM group, and about 2 weeks in the IS group. Therapy with FCM is beneficial in pregnant women at risk of postpartum hemorrhage and gynecologic patients requiring urgent surgery for uncontrolled menorrhagia.^[16]^ In a study by Koch et al^[26]^ on the ideal iron dose in IDA, 1500 mg IV iron was more effective compared with the commonly used dose of 1000 mg IV iron. Therefore, FCM is essential for rapid iron replacement in IDA.

In a meta-analysis on the safety of IV iron replacement therapy conducted by Avni et al, which included 103 RCTs, IV iron replacement was not associated with severe cardiovascular, respiratory, or neurologic adverse effects. However, infusion reactions at the IV site occurred after IV iron administration. In our meta-analysis, the incidence of adverse events was lower in the FCM group than in the IS group. This may be because the carboxymaltose shell of FCM minimizes the release of free iron, resulting in the delivery of greater amounts of iron to the tissues.^[39]^

As an adverse effect of IV iron, hypophosphatemia was noted in 2 RCTs in our meta-analysis.^[17,20]^ Van Wyck et al^[40]^ observed transient, asymptomatic hypophosphatemia in 70% of patients undergoing IV iron replacement with FCM, whereas Blazevic et al^[41]^ reported symptomatic hypophosphatemia in 4 patients receiving IV FCM and IS. IDA stimulates the transcription of fibroblast growth factor-23 (FGF23) in osteocytes, which results in hypophosphatemia due to the induction of phosphaturia.^[42]^ IV iron also induces hypophosphatemia by inhibiting the cleavage of intact FGF23. In general, hypophosphatemia is observed among 70% of gynecological patients receiving treatment for IDA.^[43,44]^ Therefore, serum phosphate levels should be monitored during IV iron replacement.

Regarding QoL and fatigue level, the findings of our meta-analysis were consistent with the studies by Van Wyck et al.^[40,43]^ This may be due to patient-friendly dosing, fewer hospital visits, and the rapid effect on Hb level associated with FCM therapy.

The choice of iron formulation is determined by the cost and convenience of administration.^[10]^ The controversy on the choice of IV iron formulation (FCM and IS) based on the lower versus higher cost^[17,20,36]^ has been highlighted previously. In general, IS requires multiple infusions that amount to added travel costs and lost-working days, compared with those with a single infusion of FCM.

In a meta-analysis by Qassim et al^[10]^ involving the comparison of IV iron formulations (IS, FCM, and iron polymaltose), there was no evidence that a single formulation was superior. This may be due to the omission of a few studies on IV IS and FCM therapies and the difference in the types of studies included (21 RCTs and 26 observational studies).

This meta-analysis had the following strengths. The included studies were restricted to RCTs, and there were no restrictions on language or type of publication. However, our review had several limitations. First, we included some blinded studies with a high risk of bias. Second, almost all the included studies were conducted in the same country. Third, there were differences in time points of measurement of Hb and ferritin levels among the studies. Fourth, the number of included studies was small, and the study population comprised women alone.

Animal studies have shown evidence of embryotoxicity and an increase in fetal skeletal abnormalities at maternally toxic doses of IV iron (“Product Information, Injectafer [ferric carboxymaltose],” American Regent Laboratories Inc, Shirley, NY). However, no published human data are available on developmental adverse outcomes associated with the use of IV FCM or IS. Therefore, there is a need for caution regarding the use of IV iron replacement for the treatment of IDA in the first trimester of pregnancy; however, IV iron replacement may be recommended in the second and third trimesters.

## 5 Conclusions

In our meta-analysis, FCM therapy showed better efficacy in increasing Hb and ferritin levels, as well as a more favorable safety profile with fewer adverse events than IS therapy in IDA among obstetric and gynecologic patients. However, our study had the limitation of a small number of included RCTs with a high heterogeneity. Therefore, further well-designed RCTs with a low risk of bias are necessary to validate our results.

## Acknowledgments

The authors thank Editage (www.editage.co.kr) for English language editing.

## Author contributions

**Conceptualization:** Hye Won Shin, Doo Yeon Go, Suk Woo Lee.

**Data curation:** Hye Won Shin, Doo Yeon Go, Yoon Ji Choi.

**Formal analysis:** Hye Won Shin, Suk Woo Lee, Hae Sun You.

**Investigation:** Hye Won Shin, Suk Woo Lee, Eun Ji Ko, Yoo Kyung Jang.

**Methodology:** Hye Won Shin, Doo Yeon Go, Hae Sun You, Yoo Kyung Jang.

**Software:** Hye Won Shin, Doo Yeon Go, Suk Woo Lee, Yoon Ji Choi, Yoo Kyung Jang.

**Supervision:** Hae Sun You.

**Validation:** Yoon Ji Choi, Eun Ji Ko, Yoo Kyung Jang.

**Writing – original draft:** Hye Won Shin, Doo Yeon Go.

**Writing – review & editing:** Hye Won Shin.

## Supplementary Material

Supplemental Digital Content
