# Supplementary material for: Comparative efficacy and safety of intravenous ferric carboxymaltose and iron sucrose for iron deficiency anemia in obstetric and gynecologic patients: A systematic review and meta-analysis
Source: Medicine (Baltimore). 2021 May 21;100(20):e24571. doi: 10.1097/MD.0000000000024571 (PMC8137003; doi:10.1097/MD.0000000000024571)
Supplement: Supplemental Digital Content [file medi-100-e24571-s001.pdf]

Supporting Information

S 1. The search strategy

Pubmed

1. Anemia[mh:noexp] OR Anemia, Iron-Deficiency[mh] OR anemia[tiab] OR anemias[tiab] OR anaemia[tiab] OR anaemias[tiab] 158012
2. ferric carboxymaltose[nm] OR ferric carboxymaltose[tiab] OR iron carboxymaltose[tw] OR iron dextri-maltose[tw] OR Ferinject[tw] OR injectafer[tw] OR VIT-45[tw] 424
3. Ferric Oxide, Saccharated[mh] OR "saccharated ferric oxide"[tw] OR "iron sucrose"[tw] OR "iron saccharate"[tw] OR "ferric saccharate"[tw] OR "ferri saccharate"[tw] OR "iron (iii) hydroxide sucrose complex"[tw] OR venofer[tw] OR hippiron[tw] OR colliron[tw] OR feriv[tw] OR fermed[tw] OR ferrinemia[tw] OR ferrisaccharate[tw] OR ferrivenin[tw] OR fesin[tw] OR iviron[tw] OR "neo ferrum"[tw] OR proferrin[tw] OR referen[tw] OR reoxyl[tw] OR sucrofer[tw] OR "xi 921"[tw] OR "ferric oxide saccharate"[tw] 884
4. #1 AND #2 AND #3 89
5. (randomized controlled trial[pt] OR controlled clinical trial[pt] OR randomized[tiab] OR placebo[tiab] OR drug therapy[sh] OR randomly[tiab] OR trial[tiab] OR groups[tiab]) NOT (animals[mh] NOT humans[mh]) 3948564
6. Women[mh] OR woman[tw] OR women[tw] OR female[tw] OR females[tw] 8661804
7. pregnant[tw] OR pregnancy[tw] OR postpartum[tw] OR prepartum[tw] OR post-partum[tw] OR pre-partum[tw] OR menorrhagia[tw] OR heavy menstrual bleeding[tw] OR hypermenorrhea[tw] OR hypermenorrhoea[tw] OR menorrhagy[tw] OR puerperium[tw] OR puerperal[tw] OR postnatal[tw] OR prenatal[tw] OR labor[tw] OR labour[tw] OR obstetric* OR gynecolog* OR gynaecolog* OR obgy OR ob-gy OR ob-gyn OR obgyn 1367198
8. (Child[mh] OR Infant[mh] OR Adolescent[mh] OR child[tiab] OR children[tiab] OR infant[tiab] OR infants[tiab] OR newborn[tiab] OR newborns[tiab] OR adolescent[tiab] OR adolescents[tiab] OR adolescence[tiab] OR teen[tiab] OR teens[tiab] OR teenager[tiab] OR teenagers[tiab] OR youth[tiab] OR youths[tiab] OR boy[tiab] OR boys[tiab] OR girl[tiab] OR girls[tiab]) NOT ((Child[mh] OR Infant[mh] OR Adolescent[mh] OR child[tiab] OR children[tiab] OR infant[tiab] OR infants[tiab] OR newborn[tiab] OR newborns[tiab] OR adolescent[tiab] OR adolescents[tiab] OR adolescence[tiab] OR teen[tiab] OR teens[tiab] OR teenager[tiab] OR teenagers[tiab] OR youth[tiab] OR youths[tiab] OR boy[tiab] OR boys[tiab] OR girl[tiab] OR girls[tiab]) AND Adult[mh]) 2178467
9. (#5 AND #6 AND #7) NOT #8 169391
10. #4 AND #9 11

EMBASE

1. anemia/de OR 'iron deficiency anemia'/exp OR (a?nemia OR an?emias):ti,ab 202,723
2. ('ferric carboxymaltose'/exp OR ('ferric carboxymaltose' OR 'iron carboxymaltose' OR 'iron dextri-maltose' OR Ferinject OR injectafer OR 'VIT-45'):ti,ab) 1,210
3. ('iron saccharate'/exp OR ('saccharated ferric oxide' OR 'iron sucrose' OR 'iron saccharate' OR 'ferric saccharate' OR 'ferri saccharate' OR 'iron (iii) hydroxide sucrose complex' OR venofer OR hippiron OR colliron OR feriv OR fermed OR ferrinemia OR ferrisaccharate OR ferrivenin OR fesin OR iviron OR 'neo ferrum' OR proferrin OR referen OR reoxyl OR sucrofer OR 'xi 921' OR 'ferric oxide saccharate'):ti,ab) 2,424
4. #1 AND #2 AND #3 367
5. ('randomized controlled trial'/de OR 'controlled clinical trial'/de OR randomi?ed:ab OR placebo:ab OR ‘drug therapy’:lnk OR randomly:ab OR trial:ab OR groups:ab) NOT (animal/exp NOT human/exp) 6,148,972
6. female/de OR (woman OR women OR female OR females):ti,ab 9,227,187
7. (pregnant OR pregnancy OR postpartum OR prepartum OR post-partum OR pre-partum OR menorrhagia OR heavy menstrual bleeding OR hypermenorrhea OR hypermenorrhoea OR menorrhagy OR puerperium OR puerperal OR postnatal OR prenatal OR labor OR labour):ti,ab OR obstetric* OR gynecolog* OR gynaecolog* OR obgy OR ob-gy OR ob-gyn OR obgyn 1,190,390
8. (child/exp OR adolescent/exp OR (child OR children OR infant OR infants OR newborn OR newborns OR adolescent OR adolescents OR adolescence OR teen OR teens OR teenager OR teenagers OR youth OR youths OR boy OR boys OR girl OR girls):ti,ab) NOT ((child/exp OR adolescent/exp OR (child OR children OR infant OR infants OR newborn OR newborns OR adolescent OR adolescents OR adolescence OR teen OR teens OR teenager OR teenagers OR youth OR youths OR boy OR boys OR girl OR girls):ti,ab) AND adult/exp) 2,685,267
9. (#5 AND #6 AND #7) NOT #8 198,665
10. #4 AND #9 27

Cochrane

1. [mh ^Anemia] OR [mh "Anemia, Iron-Deficiency"] OR (a?nemia OR an?emias):ti,ab 13813
2. ("ferric carboxymaltose" OR "iron carboxymaltose" OR "iron dextri-maltose" OR Ferinject OR injectafer OR "VIT-45"):ti,ab 361
3. [mh "Ferric Oxide, Saccharated"] OR ('saccharated ferric oxide' OR 'iron sucrose' OR 'iron saccharate' OR 'ferric saccharate' OR 'ferri saccharate' OR 'iron (iii) hydroxide sucrose complex' OR venofer OR hippiron OR colliron OR feriv OR fermed OR ferrinemia OR ferrisaccharate OR ferrivenin OR fesin OR iviron OR 'neo ferrum' OR proferrin OR referen OR reoxyl OR sucrofer OR

'xi 921' OR 'ferric oxide saccharate'):ti,ab 457

4. #1 AND #2 AND #3 50
5. [mh Women] OR (woman OR women OR female OR females):ti,ab 204629
6. (pregnant OR pregnancy OR postpartum OR prepartum OR post-partum OR pre-partum OR menorrhagia OR heavy menstrual bleeding OR hypermenorrhea OR hypermenorrhoea OR menorrhagy OR puerperium OR puerperal OR postnatal OR prenatal OR labor OR labour):ti,ab OR obstetric* OR gynecolog* OR gynaecolog* OR obgy OR ob-gy OR ob-gyn OR obgyn 92065
7. ([mh Child] OR [mh Infant] OR [mh Adolescent] OR (child OR children OR infant OR infants OR newborn OR newborns OR adolescent OR adolescents OR adolescence OR teen OR teens OR teenager OR teenagers OR youth OR youths OR boy OR boys OR girl OR girls):ti,ab) NOT (([mh Child] OR [mh Infant] OR [mh Adolescent] OR (child OR children OR infant OR infants OR newborn OR newborns OR adolescent OR adolescents OR adolescence OR teen OR teens OR teenager OR teenagers OR youth OR youths OR boy OR boys OR girl OR girls):ti,ab) AND [mh Adult]) 231705
8. (#5 AND #6) NOT #7 39325
9. #4 AND #8 14

Search No. (Duplication No.)

PubMed	11 (11)	0
EMBASE	27 (18)	9
Cochrane	14 (5)	9
Total	52 (34)	
Final reference	18	
